# Trends and Characteristics of Medication Use Among U.S. Older Adults by Frailty: A 20-Year NHANES Analysis

**DOI:** 10.1093/geroni/igaf122.4036

**Published:** 2025-12-31

**Authors:** Maryanne Kim, Michael Steinman, Matthew Growdon

**Affiliations:** University of California, San Francisco, San Francisco, California, United States; University of California, San Francisco, San Francisco, California, United States; University of California, San Francisco, San Francisco, California, United States

## Abstract

Frailty, a syndrome of reduced physiologic reserve, is associated with higher medication burden and vulnerability to adverse drug events. Reducing this burden requires understanding which therapeutic classes predominate and how their distribution varies by frailty status; however, long-term, population-based data remain limited. We analyzed data from the U.S. National Health and Nutrition Examination Survey (NHANES), 1999–March 2020, including 12,766 adults aged ≥65 years with ≥80% of frailty index (FI) components available, categorizing participants as robust, frail or severe frailty. Trends in therapeutic class use were examined across survey years. Mean medication counts rose from 2.60 to 3.52 in robust, 4.23 to 5.66 in frail, and 6.08 to 7.87 in severe frailty. Cardiovascular and metabolic agents predominated in all frailty groups, with higher counts in more frail groups. By 2017–2020, older adults with severe frailty used more than twice as many drugs from cardiovascular (2.59 vs 1.02) and metabolic (1.88 vs 0.81) classes as robust individuals. Over the 21-year period, the use of psychotherapeutic agents rose in all groups, nearly quadrupling in people with severe frailty (0.09 to 0.38), while central nervous system agents use increased minimally in robust and frail groups but meaningfully in severe frailty group (0.66 to 0.90). Medication burden increased across all frailty strata, with severe frailty showing higher use of cardio-metabolic agents and larger increase in psychotherapeutic and central nervous system agents. Medication optimization strategies for older adults should be tailored to frailty status, with attention to therapeutic classes most likely to drive polypharmacy.

